# Attentional Cueing and Executive Deficits Revealed by a Virtual Supermarket Task Coupled With Eye-Tracking in Autism Spectrum Disorder

**DOI:** 10.3389/fpsyg.2021.671507

**Published:** 2021-08-31

**Authors:** Susana Mouga, Isabel Catarina Duarte, Cátia Café, Daniela Sousa, Frederico Duque, Guiomar Oliveira, Miguel Castelo-Branco

**Affiliations:** ^1^CIBIT - Coimbra Institute for Biomedical Imaging and Translational Research, University of Coimbra, Coimbra, Portugal; ^2^Institute of Nuclear Sciences Applied to Health, University of Coimbra, Coimbra, Portugal; ^3^Center for Neuroscience and Cell Biology - Institute for Biomedical Imaging and Life Sciences, Faculty of Medicine, University of Coimbra, Coimbra, Portugal; ^4^Neurodevelopmental and Autism Unit From Child Developmental Centre, Hospital Pediátrico, Centro Hospitalar e Universitário de Coimbra, Coimbra, Portugal; ^5^Centro de Investigação e Formação Clínica, Hospital Pediátrico, Centro Hospitalar e Universitário de Coimbra, Coimbra, Portugal; ^6^Faculty of Medicine, University Clinic of Pediatrics, University of Coimbra, Coimbra, Portugal; ^7^Faculty of Medicine, University of Coimbra, Coimbra, Portugal

**Keywords:** autism spectrum disorder, attentional cueing, executive functioning, social cognition, eye-tracking, ecological task

## Abstract

Executive functioning (EF) impairments in Autism Spectrum Disorder (ASD) impact on complex functions, such as social cognition. We assessed this link between EF, attentional cueing, and social cognition with a novel ecological task, “EcoSupermarketX.” Our task had three blocks of increasing executive load and incorporated social and non-social cues, with different degrees of saliency. Performance of ASD and typical neurodevelopment was compared. The ASD showed a significant performance dependence on the presence of contextual cues. Difficulties increased as a function of cognitive load. Between-group differences were found both for social and non-social salient cues. Eye-tracking measures showed significantly larger fixation time of more salient social cues in ASD. In sum, EcoSupermarketX is sensitive to detect EF and attentional cueing deficits in ASD.

## Introduction

Autism spectrum disorder (ASD) is a neurodevelopmental disorder defined by deficits in social communication and interaction, as well as repetitive patterns of behavior and restricted interests (American Psychiatric Association, 2013). Several cognitive models (Baron-Cohen et al., 1985; Happé and Frith, 2006) have been proposed to explain the characteristics and difficulties that the individuals with ASD undergo across their life span (Lever and Geurts, 2016; Olde Dubbelink and Geurts, 2017). The general executive dysfunction hypothesis is the basis of one of these models, which suggests that complex behavioral manifestations of ASD are consequences of impaired executive processing, with empirical studies suggesting a broad impairment in executive functions (EFs) with a significant inter-individual variability (Pennington and Ozonoff, 1996; Hill, 2004a).

Some studies report that lack of cognitive flexibility or set-shifting can explain restricted and repetitive behaviors and also rigid and perseverative behaviors (Hill, 2004b; Lopez et al., 2005; McKeith et al., 2005; South et al., 2007). Others focused on social deficits, while linking these ASD characteristics with impairments in EFs, such as inhibition, information recall, flexibility, and the ability to monitor, update, and select socially appropriate responses (Channon et al., 2001; Joseph and Tager-Flusberg, 2004; Dennis et al., 2009). The EF is also associated with socialization and communication in ASD (McEvoy et al., 1993; Gilotty et al., 2002; Pellicano et al., 2006; Dichter et al., 2009; Kenworthy et al., 2009; Leung et al., 2016), and impaired EF may have a cascading impact on other social cognitive aspects, such as the development of the theory of mind (Russell et al., 1999; Jones et al., 2018), or joint attention (McEvoy et al., 1993). Faja and Dawson (2014), for instance, found that the flexibility of an individual to communicate with and respond to others, adjust social behaviors within interactional contexts, and to multi-task between processing dynamic social information and formulating an appropriate response, may be influenced by difficulties in set shifting or working memory. On the other hand, Ozonoff et al. (2004) found no significant associations between performance-based EF and social skills, but found that planning, a metacognitive skill, was associated with adaptive communication skills. On the contrary, Kenworthy et al. (2009) found that performance-based measures of improved divided attention and verbal fluency were related to fewer social symptoms. Other studies failed to find significant connections between EF and the social domain of impairment in ASD (Joseph and Tager-Flusberg, 2004; Landa and Goldberg, 2005; Cantio et al., 2016).

Impairments in EF are frequent in individuals with ASD from early ages and are thought to have a significant influence in their social cognition, adaptive behavior, and to be major contributors to everyday deficits, disability, and absence of autonomy at the most varied levels (Geurts et al., 2014; Leung and Zakzanis, 2014; Lai et al., 2017; Demetriou et al., 2018).

These deficits are attributed to atypical functional brain connectivity, with conflicting reports emphasizing either over-connectivity or under-connectivity in individuals with ASD (Maximo et al., 2014). With regard to the regions of brain impacted by ASD, the literature reports abnormalities across the default, salience, and executive control networks (Abbott et al., 2016), as well as the cortical (unimodal and supramodal brain networks) connectivity to subcortical areas, such as the thalamus and the basal ganglia (Maximo and Kana, 2019), with alterations that have been reported to persist across the life span (Braden et al., 2017).

The association between non-social and social cognitive impairments is a topic of major importance. Nevertheless, most studies exclusively focus on non-social or social cognition independently (Velikonja et al., 2019). Social attention may recruit multiple cognitive modules involved in EF and social cognition.

For these reasons, the role of EF on social cognition in individuals with ASD is still an open question in the scientific literature.

Importantly, investigation of social and non-social cognition performance may be dependent of the context where such skills are tested. Therefore, typically developing subjects search for cues in the environment that can orient to the appropriate behavior, and adjust their attentional focus and consequently result in the next action (Travers et al., 2013). This is referred to as contextual cueing, which depends on the ability to learn contingencies, associations, or probabilities that are embedded in that environment and determines the allocation of our attention to areas that provide the most relevant information for decoding complex visual inputs (Chun and Jiang, 1998). Some studies have shown that individuals with ASD demonstrate intact contextual cueing (Barnes et al., 2008; Brown et al., 2010; Kourkoulou et al., 2012), while others show that subjects with ASD have difficulties in implicitly learning the predictive relation between the location of an object and the context of other objects in the environment, but not with salient spatial cues (Travers et al., 2013).

Taken together, despite the plethora of studies, there is a strong conceptual debate in the link between EF and social/non-social cognition research that would benefit from an integrated research methodology, taking into account the ecological validity, unifying experimental approaches, and neuropsychological testing. In fact, there is need for more ecological tasks testing visual attention, using complex and dynamic stimuli that could potentially better describe the contextual factors which might affect the performance of the individuals with ASD in response to not only social stimuli, but also to non-social stimuli (Congiu et al., 2016; Liberati et al., 2017). Additionally, part of the studies in the existing literature have used archival clinical data without control groups to confirm the link between EF and ASD symptomology (Pugliese et al., 2016; White et al., 2017). These executive deficits also need to be accurately identified and clinically assessed as they can have a significant impact on the quality of life and the daily functioning of individuals with ASD (Kapp, 2018).

Moreover, such ecological contexts provide an opportunity to test the role of (social/non-social) contextual cueing in EF tasks. Our study aimed to study the link between EF and social/non-social contextual cueing in an ecological approach. To achieve the primary goal to investigate the link between EF and social cognition and the secondary goal, namely investigating the influence of social/non-social contextual cueing, we developed a task at our Lab, EcoSupermarketX, a non-immersive virtual reality task, monitored with eye-tracking, featuring a shopping task at a supermarket. EcoSupermarketX was based on two main premises: on the one hand, shopping is a good example of a real-world task that often draws heavily on EF, contextual cueing, and social/non-social cognition and on the other hand, different assessment and rehabilitation studies of ASD populations have successfully used supermarket settings (Carr and Carlson, 1993; Lamash and Josman, 2019).

We hypothesized that the subjects with ASD will show more errors, longer time, and wider distance in the EcoSupermarketX task, and deficits in attentional contextual cueing, compared to TD, and this would be associated with ASD symptom severity and with impaired EF in classical neuropsychological assessments.

## Methods

### Participants

The study included two groups of participants: the experimental group, composed by individuals with high functioning ASD; and the control group, composed by individuals with typical neurodevelopment (TD). A total of 35 participants were enrolled in the study; however, two ASD participants were excluded due to not being able to complete the task. A total of 17 participants in the ASD group (median age = 16 years and 4 months) and 16 in the TD group (median age = 15 years and 2 months) completed the protocol of the study and entered the data analysis. Groups were matched by chronological age, performance intelligence quotient (PIQ) (Jarrold and Brock, 2004), gender, and handedness (Mann–Whitney *U* or Pearson Chi-Square test, *p* > 0.05). Further groups' characterization details can be found in Table 1.

**Table 1:** Characterization of the ASD and TD groups.

	**ASD**	**TD**	
	**Median** **(IQR; min–max)**	**Median** **(IQR; min–max)**	
*N*	17	16	
Gender (M/F)	16/1	14/2	*
CA (years and months)	16 y 4 m (3 y 11 m; 12 y 11 m−22 y 4 m)	15 y 2 m (3 y 4 m; 10 y 8 m−18 y 6 m)	*
Handedness (R/L)	16/1	14/2	*
FSIQ	93.0 (19; 71–137)	116.5 (30; 92–152)	
VIQ	92.0 (20; 78–126)	120.5 (32; 91–146)	
PIQ	101.0 (19; 73–136)	107.0 (21; 85–146)	*
ADI-R RSI	16.5 (11; 7–26)	–	
ADI-R L/C	9.5 (4; 7–22)	–	
ADI-R RB/I	5.0 (4; 3–11)	–	
ADOS COM	5.0 (2; 3–7)	–	
ADOS SI	8 (4; 4–14)	–	
ADOS total	12.0 (5; 8–19)	–	

ASD, autism spectrum disorder group; TD, typical neurodevelopment group; IQR, interquartile range; min, minimum; max, maximum; M, male; F, female; CA, chronological age; FSIQ, full-scale intelligence quotient (IQ); VIQ, verbal IQ; PIQ, performance IQ; ADI-R L/C, autism diagnostic interview—revised language/communication; ADI-R RSI, ADI-R reciprocal social interactions; ADI-R RB/I, ADI-R repetitive behaviors/interests; ADOS COM, ADOS communication; ADOS SI, ADOS social interaction. *Mann–Whitney U or Pearson Chi-Square p > 0.05.

Participants with ASD were recruited from the Neurodevelopmental and Autism Unit from Child Developmental Centre, Paediatric Hospital, Centro Hospitalar e Universitário de Coimbra, Portugal. ASD diagnosis was assigned on the basis of the gold standard instruments: parental or caregiver interview—Autism Diagnostic Interview– Revised, ADI-R (Lord et al., 1994; Le Couteur et al., 2003), direct structured proband assessment—Autism Diagnostic Observation Schedule, ADOS (Lord et al., 1989; Lord and Rutter, 1999), and clinical examination performed by an experienced neurodevelopmental pediatrician, based on the current diagnostic criteria for ASD from the Diagnostic and Statistical Manual of Mental Disorders 5, DSM-5 (American Psychiatric Association, 2013). All patients with ASD had positive results in the ADI-R and ADOS for autism or ASD and met the criteria for ASD from the DSM-5. Parents also responded to Autism Behaviour Checklist, ABC (Krug et al., 1980), Social Communication Questionnaire, SCQ (Rutter et al., 2003), and Social Responsiveness Scale, SRS (Constantino and Gruber, 2005), to better characterize the behavior of participants with ASD. A comprehensive medical observation excluded associated medical condition, such as epilepsy, neurocutaneous or other genetic syndromes, or other usual comorbidities in ASD samples.

The parents of participants with TD completed SCQ (Rutter et al., 2003) and SRS (Constantino and Gruber, 2005) to exclude ASD symptomatology.

Both groups underwent an exhaustive neuropsychological evaluation and an assessment of the intelligence quotient (IQ) to exclude intellectual disabilities (all participants had a full scale IQ >70).

Written informed consent was obtained from the parents of the participants or, when appropriate, the participants themselves. Children and adolescents also gave oral informed consent. The study was approved by the ethics committees from the Faculty of Medicine from the University of Coimbra and the Centro Hospitalar e Universitário de Coimbra and was conducted in accordance with the Declaration of Helsinki.

### Procedure

In the present study, an experimental task using virtual reality stimuli, named EcoSupermarketX, was conducted. The study protocol included two different components: the EcoSupermarketX task and a neuropsychological test battery, focused on EF. During the EcoSupermarketX task, the eye movements of the participants were monitored (for technical details, see below).

#### EcoSupermarketX

EcoSupermarketX is a non-immersive virtual reality task that aims to accurately evaluate the social cognition and EF abilities of the participants using a realistic type of scenario mimicking everyday life—a computer-generated supermarket.

EcoSupermarketX is a new assessment tool created at our laboratory in order to add performance-based information to the other cognitive and executive measures used.

##### EcoSupermarketX Apparatus, Stimuli, and Design

The EcoSupermarketX stimuli were generated with Vizard Virtual Reality toolkit—version 5.2 (WorldViz, Santa Barbara, USA). The task was implemented on a desktop computer and presented in a 32-inch flat-screen with a resolution of 1,920 × 1,080 pixels, in full screen mode. After a brief summary of the task has been given, the head of the participant was immobilized via a chin and forehead support placed at the edge of the table on which the monitor was located (at a distance of ~90 cm). The participant experienced the supermarket environment from a first-hand perspective and used a joystick to navigate in the scenario.

The task included a practice block, where the participants were asked to explore the scenario of the supermarket and to familiarize with the use of joystick. Following the practice block, three different condition blocks were presented with increasing executive load (increased number of items to “buy”) and with or without cues (social, non-social, or no cue).

In the practice block, the participant had 5 min to explore the supermarket environment freely and to familiarize with the use of joystick. The joystick was adapted to right or left-handed participants that allowed them to navigate in the scenario and to rotate the scenario to the side (as if they were turning the head and looking right or left). This practice block was designed to guarantee that each participant was completely familiarized with the apparatus before the test blocks began.

**Figure 1:**
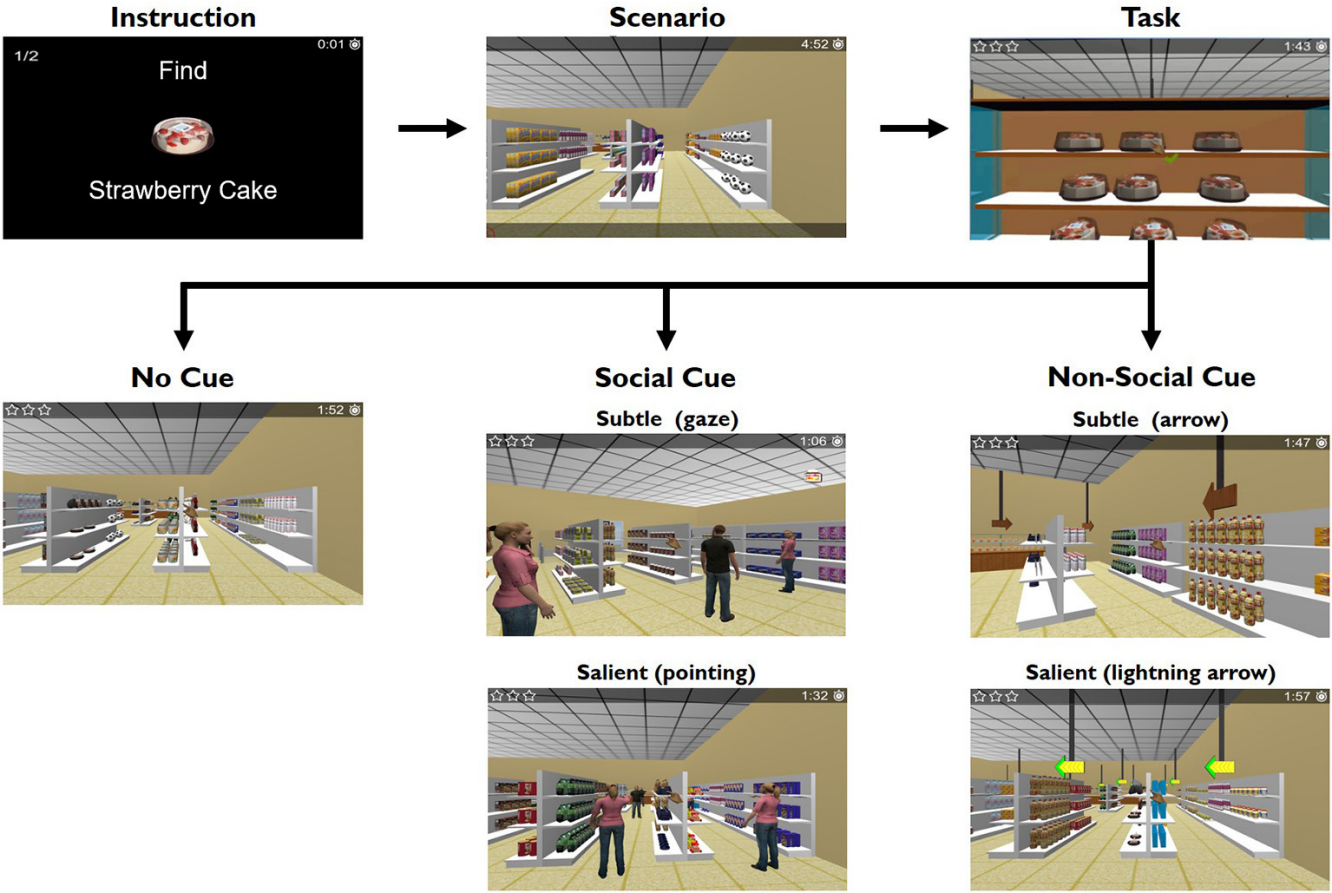
EcoSupermarketX task design, considering the different types of cues. The test blocks included an instruction that consisted in the grocery list the participants had to pick. The grocery list (2, 3, or 4-items) was presented as an instruction individually in a trial-by-trial basis: “Find strawberry cake” followed by “Find sausages” in a 2-item grocery list, for example (each item image and name appeared for 3 s). The grocery list had a variable number of items (two, three, or four) which defined the three different condition blocks with increased executive load. The groceries were replaced randomly in the shelves on a trial-by-trial basis. Participants were instructed to collect all items in the sequence they appeared in the list, and as fast and accurately as possible. Additionally, there were five different conditions in each 2, 3, or 4-item conditions: non-social salient (blinking luminous arrow), non-social subtle (wooden arrow), social salient (avatar pointing to the grocery), social subtle (avatar gazing to the grocery), and no cue.

In the test blocks, the participants were instructed to search and pick groceries from the supermarket shelves that were previous presented at a grocery list. The grocery list had a variable number of items (two, three, or four) which defined the three different condition blocks with increased executive load. The list was presented as an instruction individually in a trial-by-trial basis: “Find strawberry cake” followed by “Find sausages” in a 2-item grocery list, for example (each item image and name appeared for 3 s, see Figure 1). The groceries were replaced randomly in the shelves on a trial-by-trial basis. For every single list, which defines a trial, the participant had 1 min per item to perform the task, i.e., to find the groceries and conclude the “shopping” in a trial with a 2-items grocery list, the participant had 2 min to conclude the shopping (with a 3-item grocery list, the participant had 3 min, and 4 min to the 4-item grocery list). Participants were instructed to collect all items in the sequence they appeared in the list, and as fast and accurately as possible. They had to plan and monitor their behavior to complete the task successfully.

Additionally, participants were informed that during the task, three cueing situations could happen: they could have cueing help from a person (an avatar), an arrow or no help at all. These defined the different types of cue: social, non-social, or absent cue. They were not told what specifically the person was doing or what kind of arrows was presented, so they were not expecting different saliencies a priori. In fact, there were five different conditions in each 2, 3, or 4-item conditions: non-social salient (blinking luminous arrow); non-social subtle (wooden arrow); social salient (avatar pointing to the grocery); social subtle (avatar gazing to the grocery); and no cue (Figure 1). The blinking luminous arrow for the non-social cue and the avatar pointing to the grocery, for the social cue, are cues that are more salient, in comparison, to the wooden arrow and the avatar gazing to the grocery, respectively. The cues were not pointing directly to the target item but indicating the shortest way to find it. This sequence was maintained during all the experiments. Participants underwent five trials for the 2-item grocery list condition (one trial per cue type); 10 trials for the 3-item grocery list condition (two trials per cue type), and 15 trials for the 4-item grocery list condition (three trials per cue type), performing a total of 30 trials (with an interval between the 3 and the 4-item conditions).

EcoSupermarketX aimed to analyse executive functioning and social cognition. Therefore, to reduce mere memory constraints, an image of the requested item from the grocery list was displayed in the upper right corner after 40 s, giving the opportunity to the participant to conclude the trial. Focusing on the enhancement of the realism of the task and its ecological validity, realistic three-dimensional forms, and commercial brands were used to depict the groceries included in the supermarket scenario.

##### EcoSupermarketX Data Analysis

Several parameters were defined for the analysis of the performance of each participant in the EcoSupermarketX game, considering errors, time, distance, and head rotation variables. The different behavioral measures/parameters defined include item errors, sequencing errors, time, distance, and head rotation, which we describe below:

*Item errors*—Number of wrongly picked items of the EcoSupermarketX scenario that were not in the list of groceries divided by the number of items in the grocery list × 100 (e.g., to select a cake, when the cake was not in the list).

*Sequencing errors*—Number of picked items of the EcoSupermarketX scenario that were in the incorrect sequence according to the list of groceries divided by the number of items in the grocery list × 100 (e.g., to select sausages before cereals, when the cereals were first in the list).

*Total time—*Performance time (in seconds)—The time the participant was engaged in the execution of the trial: looking for and grabbing the products that were in the grocery list (time elapsed from the end of the grocery list memorization to the last correctly picked item).

*Total distance—*Performance distance—The distance the participant goes through in the execution of the task, looking for and grabbing of the products that are in the grocery list.

*Number of head rotations—*Sum of the number of virtual head rotations by the participant (in degrees) during the time of execution of the task. In virtual reality scenarios, head movement and orientation have been used to capture and measure macro-level attention allocation (Xi and Hamari, 2021). This parameter reflects attentional control, given the fact that in the absence of cue or in the case the participant does not use the cues presented, it is necessary to “rotate the head” to navigate the supermarket and clearly see the objects on the shelves.

### Eye-Tracking Recording and Measures

Eye movements were recorded using an infrared-emitting video-based eye tracker (EyeLink 1000 Plus, SR Research, Mississauga, ON, Canada). In terms of EyeLink tracking settings, we used mono mode and pupil corneal reflection, at a 1 K sample rate. The tracker has a reported gaze position accuracy of 0.25–0.50° and a spatial resolution of 0.05. A 9-point calibration procedure with a fixation circle was performed before each block. The participants were instructed to fixate on the circle. After the calibration, there was a validation trial to ensure the precision of the data collection. The calibration process was repeated, when necessary, until the eye achieved good mapping on all nine test positions (tracking error smaller than 1° visual angle). As participants were performing a dynamic virtual-reality task in which they were freely walking around a supermarket, the frames in the screen were always different for all participants. In this way, the AOI were defined in the virtual-reality software, which received the gaze coordinates of the participants from the eye tracking software in a real-time mode. Using those screen coordinates, we computed the time that the participant was looking to each AOI in a real time basis. The areas of interest were related to the different types of cues defined: arm (while avatar is pointing), head (while avatar is looking), salient arrow, and subtle arrow.

### Neuropsychological Assessment

In addition to the assessment of intelligence quotients with the Weschler scales, we used a standard neuropsychological test battery as a baseline characterization of the executive status of the study participants. The tests were several classic executive tests widely employed in clinical and research settings, some of them included in the Coimbra Neuropsychological Assessment Battery (BANC) (Simões et al., 2016) and other classical tests. The tests selected for our study were individually administered and focused on the evaluation of executive functions, namely Corsi Blocks, which assesses visuospatial short-term memory and spatial attention; Trail, that assesses attention, processing speed, and cognitive flexibility; Tower, which assesses the executive functions of planning, working memory, rule learning, the ability to inhibit responding, self-monitoring, and regulation and problem solving. A used classical test that is not in the BANC is the Stroop color-word test (SCWT) (Stroop, 1935)—naming, reading and interference tasks, which also assess the cognitive flexibility and processing speed.

### Data Analysis and Statistics

Initially we conducted a descriptive data analysis to summarize the data using graphical techniques and quantitative analysis in order to characterize the sample, detect possible extreme outliers, and measurement error.

Non-parametric statistics were carried out for all statistical analyses to avoid biases due to deviations from normality and variance heterogeneity.

Categorical and nominal values are expressed as frequencies, and continuous data are presented as median, interquartile range (IQR), and range.

To verify the main effect of the number of items, the Jonckheere-Terpstra test was used, determining if the different behavioral measures/parameters defined (item errors, sequencing errors, total time, total distance, and head rotation) results significantly increased with the increase in the number of items. The effect of number of items in the groups was also assessed, comparing the quantitative variables in the 2-, 3-, and 4-item conditions, between the two groups (ASD and TD) using Mann–Whitney *U*-tests. The effect of cue in the groups was assessed, resorting to Mann–Whitney *U*-tests comparisons of quantitative variables between the two groups (ASD and TD), first verifying if there were differences in the distribution of the different variables of the EcoSupermarketX in the cue vs. no cue conditions. After identification of this main effect, planned analyses were then included: social vs. non-social cue, followed by subtle vs. salient, in both social and non-social cues.

Spearman's rank correlation coefficients were calculated, in the clinical group, to examine the associations of EcosupermarketX behavioral parameters, eye-tracking measures, and the neuropsychological tests referred to above, as well and the ASD core social interaction features. Benjamini–Hochberg corrections with false positive rate established at 0.05 were used to deal with multiple comparisons, and only the correlations that survived these corrections are reported in the “Results” section and further examined in the “Discussion” section.

Differences in the eye-tracking measures were assessed, resorting to Mann–Whitney *U*-tests comparisons of quantitative variables between the two groups (ASD and TD).

Effect sizes (Kendal's tau b for Jonckheere-Terpstra test statistics and Cohen's *d* for Mann–Whitney *U*-tests) are reported with *p*-values for significant statistical differences.

All outliers were considered to be clinically and scientifically relevant, and therefore we decided not to exclude them from our main analyses presented in this paper, which is justified by the use of non-parametric statistics.

All statistical analysis was completed with the support of the Statistical Package for Social Sciences, version 26 (SPSS®, Chicago, IL, USA). A significance level of 0.05 was adopted.

### Ethics Statement

All the procedures in this study were reviewed and approved by the ethics committees from the Faculty of Medicine from our University (CE-11/2013) and our Hospital (CHUC-102-13) and was conducted in accordance with the 1964 Declaration of Helsinki and its later amendments or comparable ethical standards. Written informed consent was obtained from the parents/guardians of all participants or, when appropriate, the participants themselves. Children and adolescents also gave oral informed consent.

## Results

### EcoSupermarketX

The several behavioral measures extracted from the performance of the participants in the EcoSupermarketX task gave us important indications about how well the participants with ASD could perform a task that mimics daily-life routines and is very demanding in terms of EF and social cognition. Moreover, the analysis of the EcoSupermarketX data gave us relevant information about the impact of increasing executive load and various cues on the behavior of ASD and TD populations. We defined four main categories for the report of our study: effect of cognitive load (number of items), main effect of type of cue (no cue vs. cue), and then planned analyses on cue subtypes (social vs. non-social cue, subtle vs. salient cue), eye-tracking measures, and correlation patterns.

#### Effect of Cognitive Load

**Figure 2:**
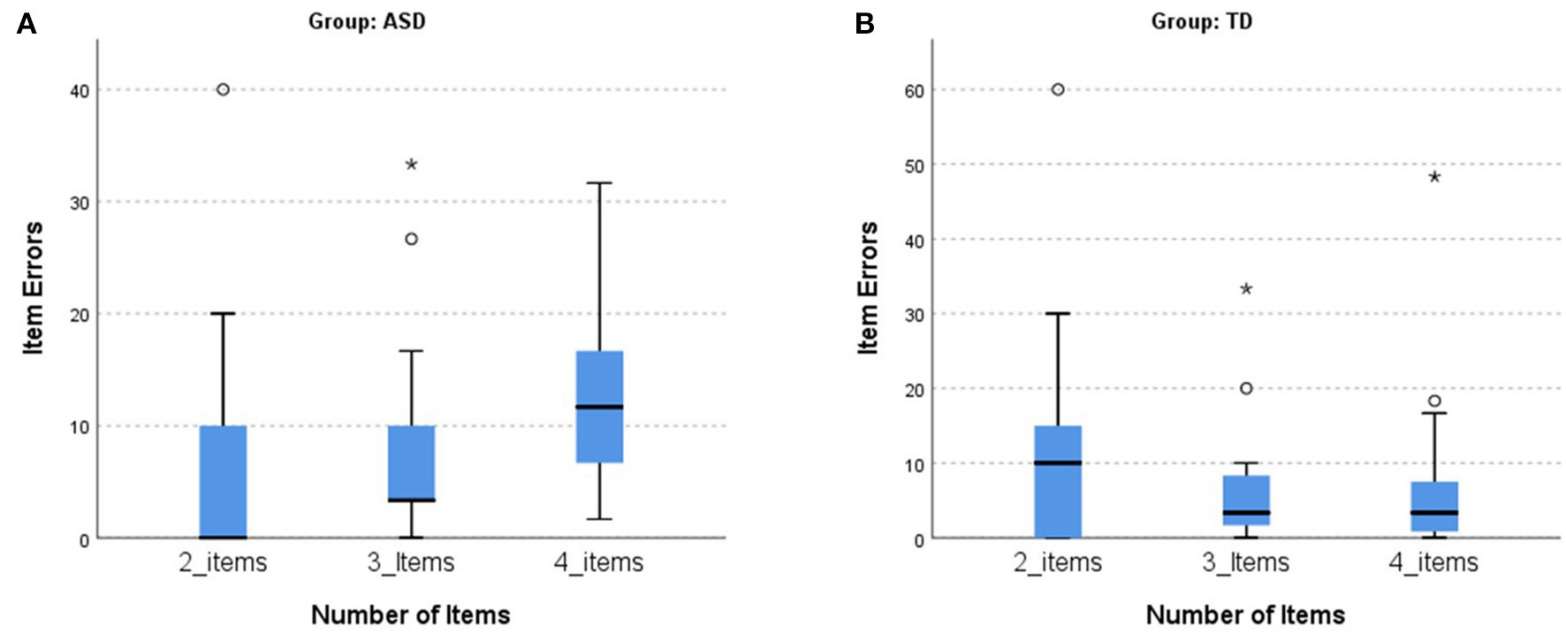
Trend analysis of the number of items: item errors. **(A)** Association between the number of item errors with the increasing number of items in the ASD group. The number of item errors is higher with the increase of cognitive load (with higher number of items per condition) in the ASD group. **(B)** No association between the number of item errors with the increasing number of items in the TD group. In the TD group, the number of item errors is similar in the different conditions, despite the increasing number of items (cognitive load). ASD, autism spectrum disorder group; TD, typical neurodevelopment group. The symbol * is the representation of an extreme outlier.

A Jonckheere-Terpstra test for ordered alternatives showed that in the ASD group, there was a statistically significant increase of item errors with increasing cognitive load (i.e., number of items in the grocery list, from “2-item” and “3-item” to “4-items”), T_JT_ = 576.50, *z* = 2.497, *p* = 0.013, Kendall's tau *b* = 0.286, which was not present in the TD group (Figure 2).

**Figure 3:**
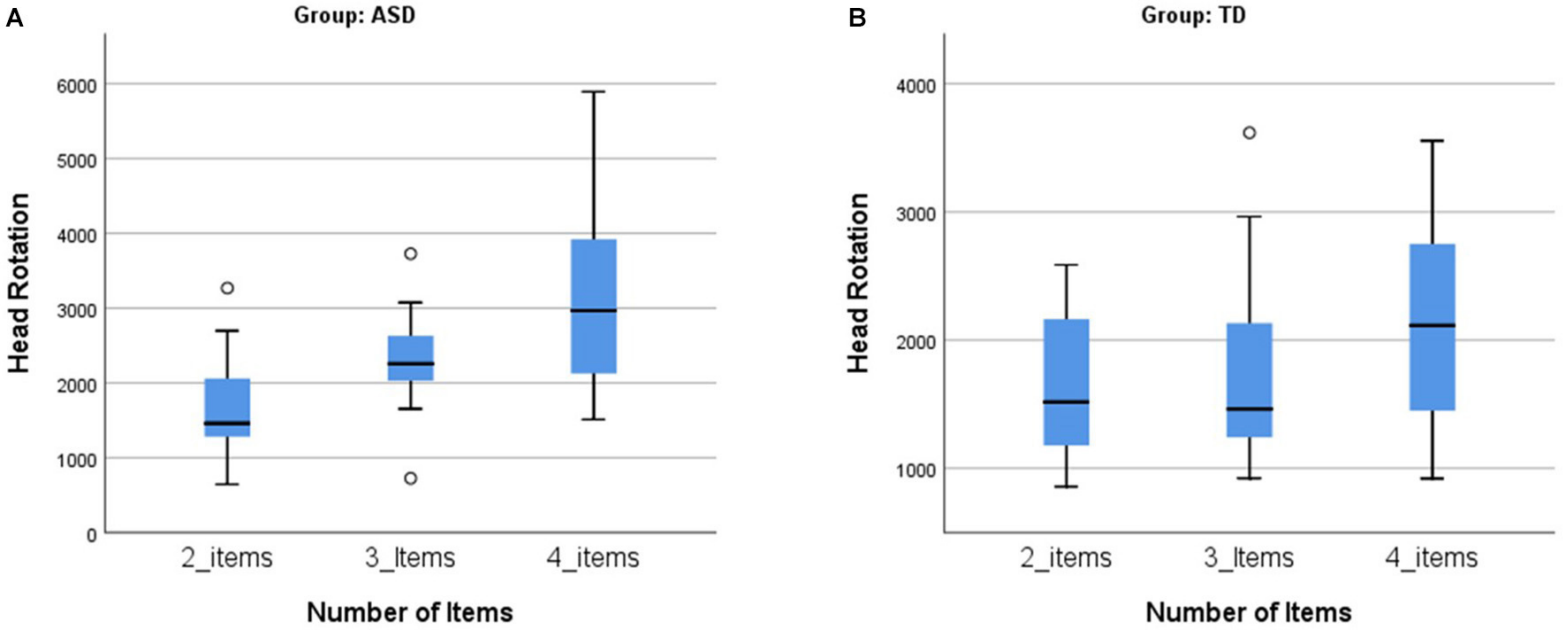
Trend analysis of the number of items: head rotation. **(A)** The association between the head rotations with the increasing number of items in the ASD group. The head rotations increase with the increment of cognitive load (with higher number of items per condition) in the ASD group. **(B)** No association between the numbers of head rotations with the increasing number of items in the TD group. In the TD group, the head rotations are similar in the different conditions, despite the increasing number of items (cognitive load). ASD, autism spectrum disorder group; TD, typical neurodevelopment group.

In the ASD group, there was a statistically significant increase of the head rotation (reflecting orienting) parameter with the increasing number of items (from “2-items” and “3-items” to “4-items”), T_JT_ = 666.00, *z* = 4.018, *p* < 0.001, Kendall's tau *b* = 0.442, which was not present in the TD group (Figure 3).

The effect of cognitive load between the groups was also assessed comparing the quantitative variables between the two groups (ASD and TD) in the three conditions: 2-items, 3-items, and 4-items conditions. In the 2-items condition we did not found differences between groups (*p* > 0.05 in all parameters). In the 3-items condition, we only found statistically significant differences in the head rotation, with ASD group (Mdn = 2,256.39) having larger values than the TD group (Mdn = 1,463.19), *U* = 70.00, *p* = 0.017, and *d* = 0.909. In the 4-items condition, we found statistically significant differences in item errors, sequencing errors, time, and head rotation. In the item errors, the ASD group (Mdn = 11.67) had higher values than the TD group (Mdn = 3.33), *U* = 64.50, *p* = 0.009, and *d* = 1.003; concerning the sequencing errors, these were statistically significantly higher for the ASD group (Mdn = 5.00) than the TD group (Mdn = 1.67), *U* = 71.50, *p* = 0.019, and *d* = 0.884. The same pattern is present in the total time, with the ASD group (Mdn = 53.91) taking more time to perform the task in the 4-items condition than the TD group (Mdn = 42.70), *U* = 71.00, *p* = 0.019, and *d* =0.893. The number of head rotations were statistically significantly higher for the ASD group (Mdn = 2965.11) than the TD group (Mdn = 2115.00), *U* = 73.00, *p* = 0.023, and *d* = 0.860.

#### Effect of Cue

The effect of cue in the groups was assessed comparing the two groups (ASD and TD) in this order: first verifying if there were differences as function of presence vs. absence of a cue. Then follow-up analyses were performed concerning social vs. non-social cue, followed by subtle vs. salient in both social and non-social cues.

**Figure 4:**
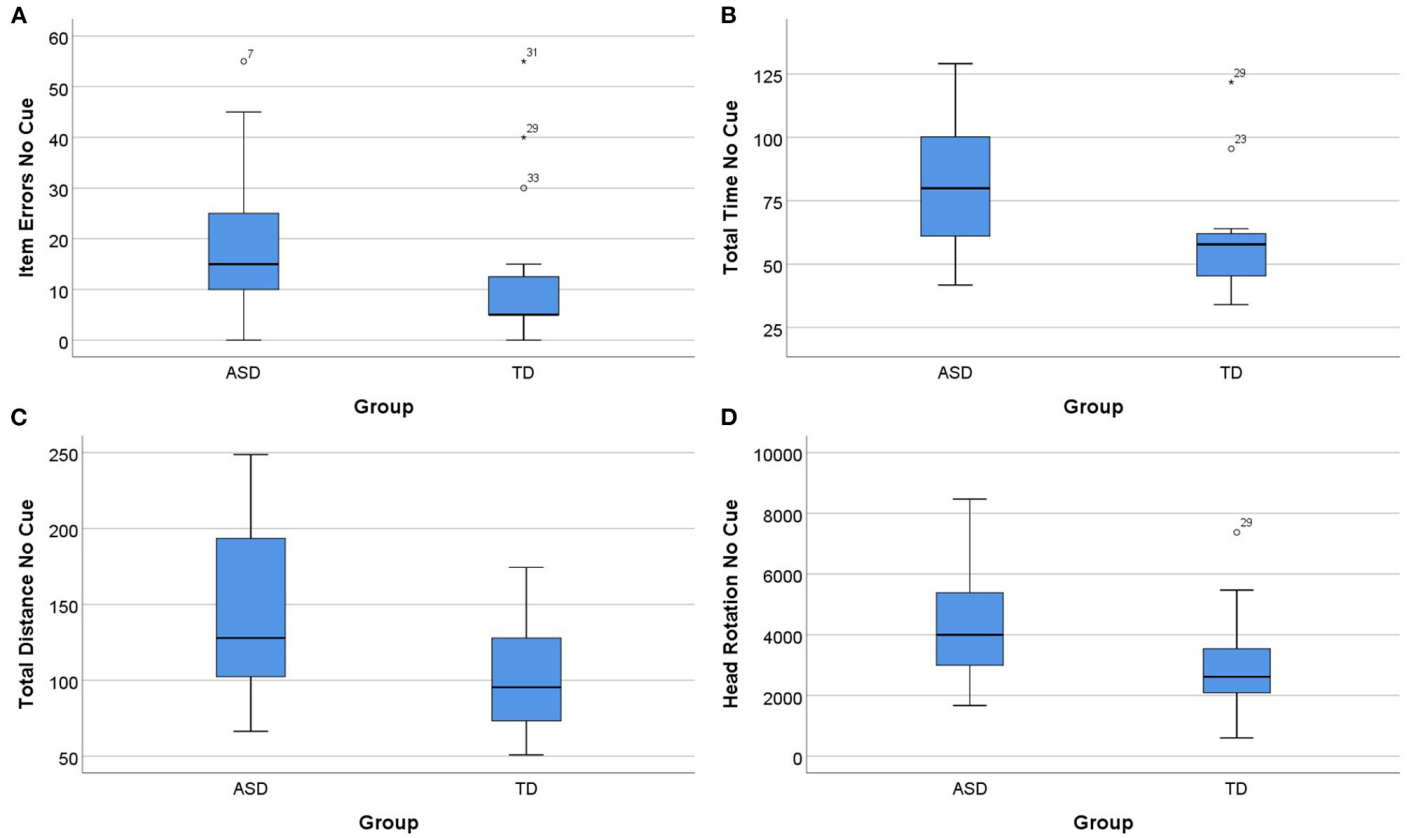
Significant group differences were observed in the no cue condition (*p* < 0.05). **(A)** Mean percentage of item errors for ASD and TD groups for the no cue condition. **(B)** Total time for ASD and TD groups for the no cue condition. **(C)** Total distance for ASD and TD groups for the no cue condition. **(D)** Head rotation for ASD and TD groups for the no cue condition. Boxplots: central mark—median; edges of box−25th and 75th percentiles; whiskers—most extreme data points (minimum and maximum). ASD, autism spectrum disorder group; TD, typical neurodevelopment group. The symbol * is the representation of an extreme outlier.

Concerning the cue factor, a Mann–Whitney *U*-test indicated differences between the groups (ASD and TD) specifically for the no cue condition, which was replicated across parameters concerning item errors, total time, total distance, and head rotation, suggesting that cue absence is very detrimental in ASD. Accordingly, item errors were statistically significantly higher for the ASD group (Mdn = 15.00) than the TD group (Mdn = 5.00), *U* = 67.50, *p* = 0.012, and *d* = 0.951. The same pattern was present in the total time, with the ASD group (Mdn = 79.90) taking more time to perform the task in the no cue condition than the TD group (Mdn = 57.78), *U* = 70.00, *p* = 0.017, and *d* =0.909. Total distance was also greater for the ASD group (Mdn = 127.87) than for the TD group (Mdn = 95.35), *U* = 81.00, *p* =0.049, and *d* = 0.735. The number of head rotations were statistically significantly higher for the ASD group (Mdn = 3997.20) than the TD group (Mdn = 2616.05), *U* = 67.00, *p* = 0.012, and *d* = 0.960. These results are summarized in Figure 4. In the parameter sequencing errors in the no cue condition and in all the parameters in the cue condition, we did not find differences between groups (*p* > 0.05).

**Figure 5:**
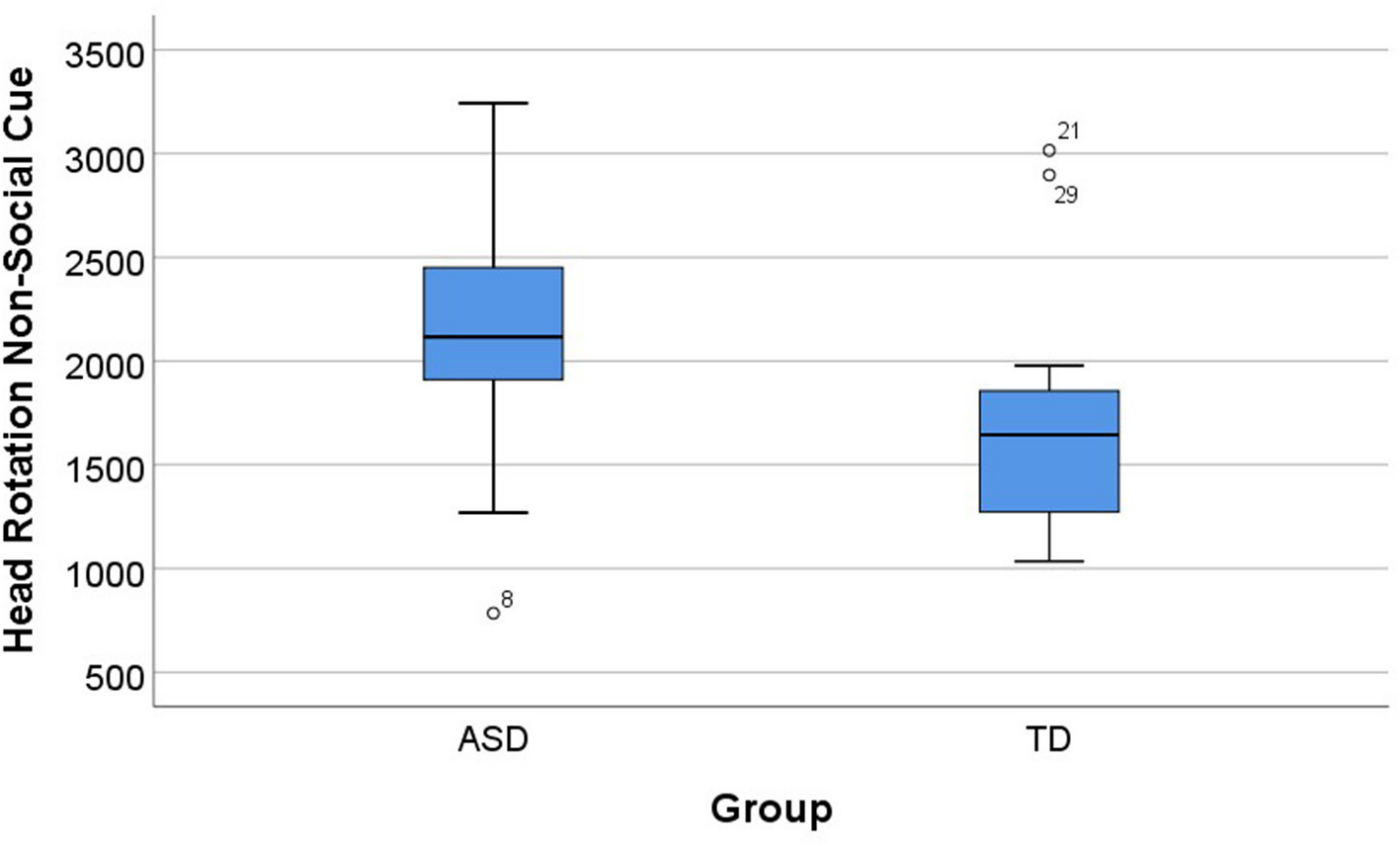
Significant group differences were observed in the non-social cue condition (*p* = 0.021). Head rotation for ASD and TD groups for the non-social cue condition. Boxplots: central mark—median; edges of box−25th and 75th percentiles; whiskers—most extreme data points (minimum and maximum). ASD, autism spectrum disorder group; TD, typical neurodevelopment group.

Even in the presence of a cue, significant group differences were observed. Concerning non-Social cue, a Mann–Whitney *U*-test indicated differences between the groups (ASD and TD) in this condition, specifically concerning number of head rotations. The number of head rotations were statistically significantly higher for the ASD group (Mdn = 2115.78) than the TD group (Mdn = 1644.09), *U* = 72.00, *p* = 0.021, and *d* = 0.876 (Figure 5). No other significant differences were found in the other parameters (item errors, sequencing errors, total time, and total distance). In the social cue condition, we did not find differences between the groups (*p* > 0.05).

**Figure 6:**
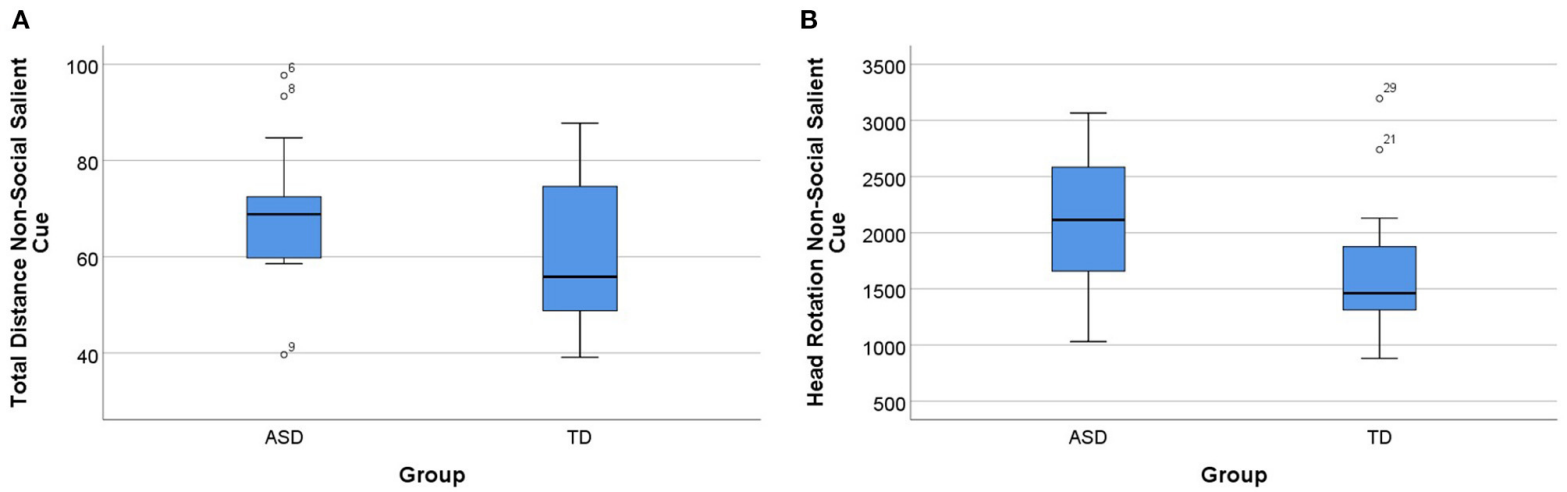
Group differences in the non-social salient cue condition. **(A)** Total distance for ASD and TD groups for the non-social salient cue condition. **(B)** Head rotation for ASD and TD groups for the non-social salient cue condition. Boxplots: central mark—median; edges of box−25th and 75th percentiles; whiskers—most extreme data points (minimum and maximum). ASD, autism spectrum disorder group; TD, typical neurodevelopment group.

We also compared the performance of both the groups (ASD and TD) in different types of saliency of the cues. It turned out that when cues were salient, significant group differences were present, but not for subtle cue types (*p* > 0.05). The Mann–Whitney *U*-test indicated differences between the groups (ASD and TD) in the non-social salient condition, specifically in total distance and head rotation. Total distance was statistically significantly higher for the ASD group (Mdn = 68.84) than the TD group (Mdn = 55.85), *U* = 81.00, *p* = 0.049, and *d* = 0.735. The same pattern was present in the number of head rotations, with the ASD group (Mdn = 2114.30) going astray in the task in the non-social salient condition, contrary to the TD group (Mdn = 1462.23), *U* = 78.00, *p* = 0.037, and *d* = 0.781. In this condition, in the other parameters (item errors, sequencing errors, and total time), we did not find differences between groups (*p* > 0.05). These results are summarized in Figure 6.

**Figure 7:**
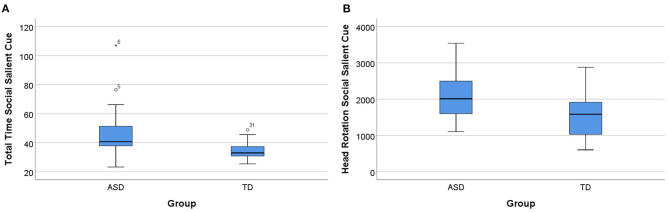
Group differences in the social salient cue condition. **(A)** Total time for ASD and TD groups for the social salient cue condition. **(B)** Head rotation for ASD and TD groups for the social salient cue condition. Boxplots: central mark—median; edges of box−25th and 75th percentiles; whiskers—most extreme data points (minimum and maximum). ASD, autism spectrum disorder group; TD, typical neurodevelopment group. The symbol * is the representation of an extreme outlier.

Concerning the social cue salient condition, the Mann–Whitney *U*-test indicated differences between the groups (ASD and TD) in this condition, specifically in the total time and head rotation. Total time was statistically significantly higher for the ASD group, with the ASD group (Mdn = 40.71), taking more time to perform the task in the social salient cue condition than the TD group (Mdn = 32.98), *U* = 78.00, *p* = 0.037, and *d* = 0.781. Head rotation was also statistically significantly higher for the ASD group (Mdn = 2,010.49) than the TD group (Mdn = 1,582.92), *U* = 81.00, *p* = 0.049, and *d* = 0.735. In this condition, in the other parameters (item errors, sequencing errors, and total distance), we did not find differences between groups (*p* > 0.05). These results are summarized in Figure 7.

#### Eye-Tracking Measures

The time looking at the different AOIs of social or non-social relevance (arm, head, salient arrow, and subtle arrow) that were related to the different types of cues (social salient, social subtle, non-social salient, and non-social subtle, respectively) was compared between the two groups (ASD and TD).

**Figure 8:**
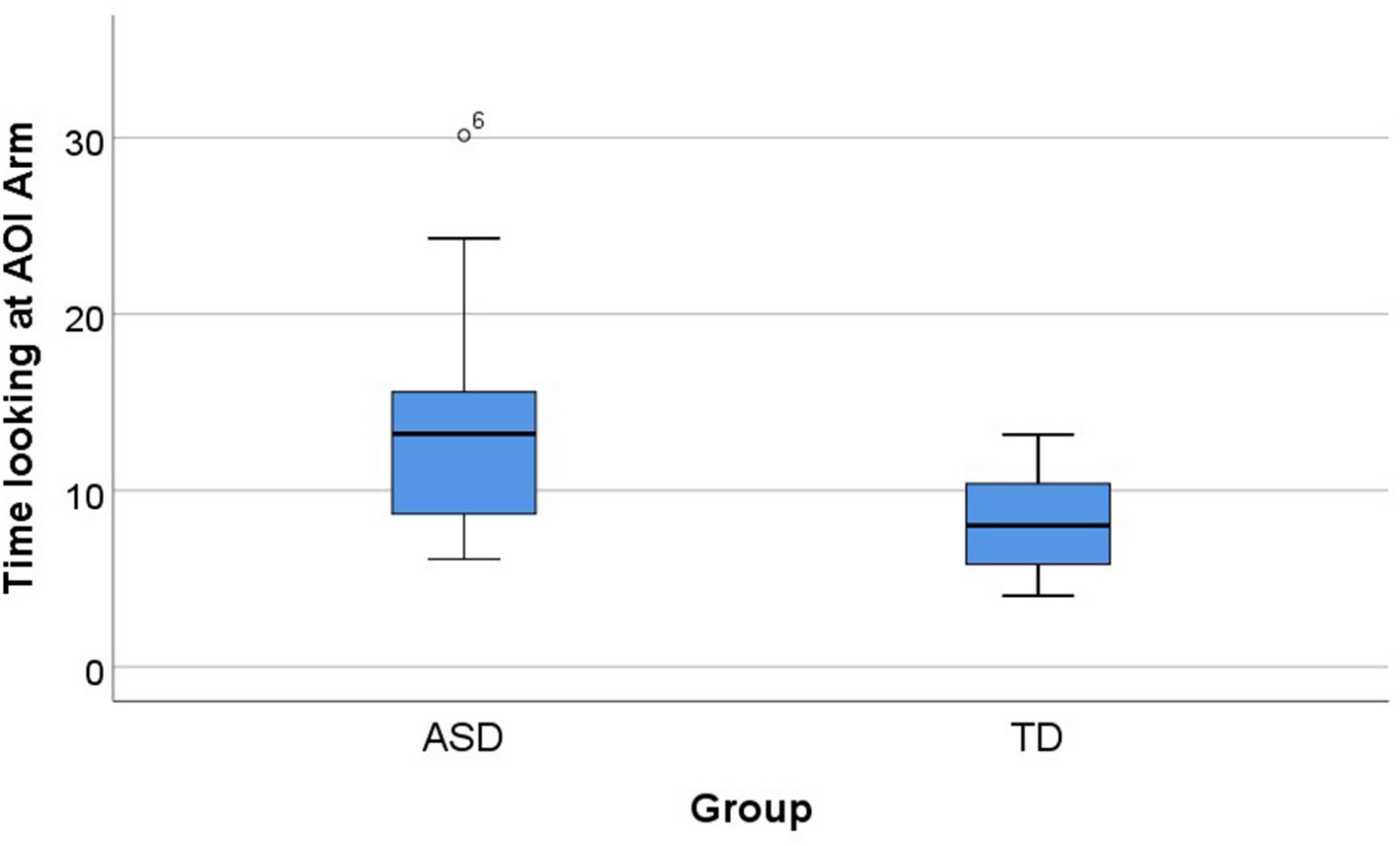
Group differences in the AOI arm (*U* = 64.00, *p* = 0.009, *d* = 1.012). Total time for ASD and TD groups in the AOI arm in the social salient cue condition. Boxplots: central mark—median; edges of box−25th and 75th percentiles; whiskers—most extreme data points (minimum and maximum). AOI, area of interest; ASD, autism spectrum disorder group; TD, typical neurodevelopment group.

The Mann–Whitney *U*-test indicated differences between the ASD and TD groups in the AOI Arm, that is presented in the social salient condition, with the ASD group (Mdn = 13.22), looking longer than the TD group (Mdn = 8.01) in the AOI Arm, *U* = 64.00, *p* = 0.009, and *d* = 1.012 (Figure 8). In the other AOIs (salient arrow, subtle arrow, and head), in the other parameters (item errors, sequencing errors, and total time), we did not find differences between groups (*p* > 0.05).

#### Correlation Analysis

We focused our correlational analyses between the behavioral measures/parameters of the EcoSupermarketX and the results of neuropsychological tests, diagnostic parameters, and eye-tracking measures that were related with our hypothesis of a relation with executive processing and the role of attention and social cues, specifically in ASD (see Table 2 for details on exact *p*-values and specific correlations).

**Table 2:** Spearman's rank correlation coefficients between the EcosupermarketX behavioral parameters, neuropsychological tests, ASD core social interaction features, and eye-tracking measures in the ASD group.

	**Corsi blocks**	**Tower** **(total of trials)**	**Tower** **(total of errors)**	**Stroop test**	**Trail flexibility index**	**RSI** **(ADI-R)**	**Time looking at AOI Arm**	**Time looking at AOI salient arrow**
Item errors (4-item)	−0.379	0.313	0.310	−0.260	0.631^*^	0.218		
Number of head rotations (4-item)	−0.594	0.459	0.430	−0.196	0.351	0.323		
Item errors (no cue)	−0.137	−0.078	−0.056	−0.177	0.172	0.119		
Total time (no cue)	−0.658^*^	0.552^*^	0.568^*^	−0.377	0.375	0.646^*^		
Total distance (no cue)	−0.568^*^	0.587^*^	0.612^*^	−0.634^*^	0.271	0.669^*^		
Number of head rotations (no cue)	−0.571	0.339	0.333	−0.428	0.354	0.607		
Number of head rotations (non-social cue)	−0.282	0.102	0.058	0.204	0.130	−0.148		
Total distance (non-social salient cue)	−0.009	0.186	0.155	0.194	0.077	−0.142		0.311
Number of head rotations (non-social salient cue)	−0.236	0.115	0.035	0.188	−0.050	0.167		−0.365
Total time (social salient cue)	−0.194	0.252	0.276	0.085	0.278	0.537	0.650^*^	
Number of head rotations (social salient cue)	−0.235	0.189	0.167	0.187	0.055	0.103	0.120	

Benjamini–Hochberg corrections with false positive rate established at 0.05 were used to deal with multiple comparisons. Correlations signaled with* are significant. RSI-ADI-R, reciprocal social interactions from autism diagnostic interview-revised; AOI, area of interest. Spearman's rank correlation;

* *p < 0.05*.

##### Cognitive Load

The cognitive load effect was evident in the total number of errors and in the number of head rotation, as number of items increases.

In the ASD group, the item errors in the 4-item condition were significantly correlated with the trail flexibility index (*r*_*s*_ = 0.63, *p* = 0.033). This was a positive correlation, which means that more errors in the EcoSupermarketX in the 4-item condition were associated with higher scores in the trail flexibility index (where higher results in the index, means lower cognitive flexibility).

In what concerns to the number of head rotations, no significant correlations after correction were found.

##### Type of Cue

The no cue condition was the one that mostly differentiated participants across parameters (see above) and the one where a larger pattern of significant correlations was found.

*No cue condition.* In the ASD group, in what concerns time, in the no cue condition, we found significant positive correlations with scores from tower [total of trials (*r*_*s*_ = 0.55, *p* = 0.033), and total errors (*r*_*s*_ = 0.57, *p* = 0.033)] and reciprocal social interaction from ADI-R (*r*_*s*_ = 0.65, *p* = 0.021), and a negative correlation with Corsi Blocks (*r*_*s*_ = −0.66, *p* = 0.021). In addition, more difficulties in social interaction are associated with more item errors in the no cue condition.

In this condition, regarding total distance, the ASD group showed significant correlations with Stroop-Interference (*r*_*s*_ = −0.63, *p* = 0.018), Corsi Blocks (*r*_*s*_ = −0.57, *p* = 0.021), tower [total of trials (*r*_*s*_ = 0.59, *p* = 0.020), and errors (*r*_*s*_ = 0.61, *p* = 0.018)] and reciprocal social interaction level from ADI-R (*r*_*s*_ = 0.67, *p* = 0.018). With tower results (total of trials and errors) and reciprocal social interaction from ADI-R, we found positive correlations, while in the Stroop test and Corsi Blocks we found negative correlations, repeating a pattern present in the previous parameter (time). In what concerns to the item errors and number of head rotations in the no cue condition, no significant correlations after correction were found.

*Non-social cue condition.* In the non-social cue, no significant correlations after correction were found between EcoSupermarketX parameters (head rotation), neuropsychological test results, diagnostic parameters, and eye-tracking measures.

*Non-social salient cue.* No significant correlations after correction were found between EcoSupermarketX parameters (distance and head rotation), neuropsychological test results, diagnostic parameters, and eye-tracking measures, in the non-social salient cue.

*Social salient cue condition.* In what concerns time, in the social salient cue condition, in the ASD group, we found significant positive correlations with the eye-tracking measure (time looking at AOI Arm) (*r*_*s*_ = 0.65, *p* = 0.033). In the social salient cue (head rotation), no significant correlations after correction were found.

## Discussion

In this study, we investigated the link between executive functions, attentional contextual cueing, and social cognition in subjects with ASD. For that purpose, we compared the performance in a novel ecological task aimed at assessing executive functioning in a daily living chore: shopping in a supermarket, with the integration of attentional social vs. non-social cues, in two matched groups of adolescents and young adults with ASD or TD. In order to answer our research question, we used markers of EF under distinct task constraints, with explicit manipulations of levels of cognitive load and attentional saliency of the social and non-social cues that could help in the performance of the task.

We found that ASD subjects are more affected with the increasing cognitive load of information, since they presented a significant increase of the item errors and head rotations with the increase in the number of items appearing in the grocery list. A higher value for the number of head rotations means that ASD participants struggled to find the right item, looking around (orienting) more and consequently rotating their “virtual head” during the task. This suggests a deficit in the efficient deployment of attention, leading to a larger number of head turns.

Cognitive load refers to the used amount of working memory resources and is thought to be a crucial factor in the learning of complex tasks (Paas et al., 2003), such as our daily living chores. Working memory is the ability to temporarily store and manipulate information; it is limited and varies from person to person (O'Hare et al., 2008; Baddeley, 2010). Working memory is also considered an essential element of cognitive control (Engle et al., 1999; Baddeley, 2010), with a critical importance for learning and academic achievement (Alloway, 2009), as well as social competency (Dennis et al., 2009). Our results, in which the ASD group presented more difficulties when the list of groceries have a higher number of items, were, therefore, in line with previous literature that report that compared to TD, individuals with ASD performed significantly worse on complex tasks related to working memory (Bennetto et al., 1996; Russell et al., 1996; Minshew and Goldstein, 2001; Ozonoff and Strayer, 2001; Williams et al., 2006). In fact, subjects with ASD seemed to present difficulties in performing the task when the difficulty increases, and this does not happen in the TD group. This corroborates other studies that have also found that when performing working memory tasks of increasing complexity or cognitive load, children with ASD were impaired compared to TD children (Minshew and Goldstein, 2001; Williams et al., 2006; Russo et al., 2007).

In what concerns to the introduction of a cue as a helping feature in the task, it seems to have an important and clarifying role in the way ASD people allocate their attention in structured environments. We found that in the absence of a cue, ASD subjects perform worse and only the addition of some types of attentional cues can rescue the impaired performance of the TD individuals. In fact, our ASD participants presented more errors, took more time to perform the task, “walked” longer distances, and were more adrift in the no cue condition, when nothing would guide their actions. This reinforces that the use of some cues seems to have a beneficial effect in restoring to the overall performance.

Furthermore, the ASD showed impairment specifically for salient cues, regardless of being social or non-social, which is surprising because one would at the first sight expect an effect for subtle cues. The Enactive Mind theory stresses that cognition is embedded in experiences resulting from the actions of body upon salient aspects of its surrounding environment and that social functioning is supported by the ability to visually track socially salient information within interactions (Klin et al., 2003). Interestingly, our ASD group spent more time looking to the AOI arm (salient social cue). They seem to take more time to interpret the environment and decide what to do than TD. This is consistent with the notion that individuals with ASD might be blind at social cues, such as gaze or gestures. For this reason, an arm pointing to a specific area of the environment might not cue their attention as in the participants with TD. Rather, their attention is “sticky” to the arm because the pointing is not meaningful itself. As a consequence, it takes more time for them to interpret the environment and to decide what to do. These results are in line with a previous study that found that the individuals with ASD have a tendency to spread their attention when exploring social cues (Liberati et al., 2017).

The significant associations found between EcoSupermarketX parameters, and the different neuropsychological assessments indicate that functional domains related to attention and EF are captured by the measures computed from this novel ecological task. Additionally, the significant correlations found between EcoSupermarketX performance and ASD core symptomatology severity give important indications about the impact on social cognition/skills and the functional implications of ASD clinical phenotype to daily living functional abilities, going beyond the previous reports (Joseph and Tager-Flusberg, 2004; Landa and Goldberg, 2005; Cantio et al., 2016).

While our results support one of the key cognitive theories of ASD, the executive dysfunction (Pennington and Ozonoff, 1996; Hill, 2004a), they also stress the importance of the of attentional contextual cueing and raises questions about the nature and influence of cue saliency. We showed that the subjects with ASD have a deficit in the allocation of attention that seems to interact with the more general deficit in EF. On the other hand, our study reports deficits not only in non-social, but also in hot EFs and cognitive processes, which represent goal-oriented behaviors (Zelazo and Carlson, 2012; Kouklari et al., 2017). This emphasizes the knowledge that ASD is not characterized by one main cognitive deficit but instead by impairments in a selective range of higher-order cognitive abilities, including attention and EF, corroborating a multiple-deficit account.

The attentional contextual cueing as a possible explanation for the difficulties in complex cognitive domains in spite of largely preserved visual spatial abilities in ASD, has been studied mostly in classic spatial-learning tasks, but much less so in the context of real life constraints and daily life chores. Some studies have suggested proficient implicit contextual cueing in individuals with ASDs as compared to participants with TD (Barnes et al., 2008; Brown et al., 2010; Kourkoulou et al., 2012; Travers et al., 2013). This is in part corroborated by our study when we show that the performance of ASD group matches the TD in the presence of the cue (showing differences in the absence of cues). The contextual cueing deficit is demonstrated by the dependence on the presence of a cue. Nonetheless, we found a surprising result in what concerns to the saliency of the cues: salient cues did not rescue performance in ASD comparing to TD, contrary to subtle cues. Brown et al. (2010) have hypothesized that ASD problems in real-world areas expected to require implicit acquisition, such as social cognition, in spite of preserved implicit learning mechanisms, may be explained by interference due to abnormal attention or the overuse of explicit strategies. We substantiate this hypothesis in our eye-tracking result. In fact, the subjects with ASD show longer fixations than TD only in the social salient cue, which is associated with worse results in the social salient condition in the task and can explain the difficulty in the daily life, where we are constantly and continuously exposed to explicit salient cues in a wide range of activities that we have to perform.

To the best of our knowledge, this is the first study that so far assesses attentional cueing and EF with social and non-social cues with different saliencies in an ecologic daily living chore context. The present findings help improve our understanding of the patterns of cognitive impairments in adolescents and young adults with ASD because we showed an impairment in ASD both in the presence of social and non-social contextual cues in an ecological task, capable to identify ASD deficits in EF and attentional cueing. The discrepancy between what ASD individuals can do on explicit tasks of non-social and social reasoning (when they receive specific instructions and all the task is compartmentalized), and what they are unable to do in the daily social life, when they have to apply spontaneously their abilities in a naturalistic situation, remains one of the most intriguing questions in this research field. As our previous works stated, even individuals with normal or high IQ cannot use their cognitive abilities to face the demands of daily living and social situations (Mouga et al., 2015, 2016). Our present study shows that attention deficits can be rescued by guiding goal-directed actions using explicit cues and stresses the importance of the structured or not structured context of the task and the cognitive load that implies. Taken together, our results point to the fact that attentional allocation in the population with ASD is context and task dependent, which extends our previous work (Bernardino et al., 2012) on contextual dependency of local vs. global attentional allocation. It also shows that cognitive load may have a large impact even on these relatively simple tasks. These results are relevant for the selection of interventional strategies in subjects with ASD, focused on the improved attentional allocation to social and non-social cues (diminishing the need to spend more time on social attention cues, such as the arm of an avatar). They also motivate future work exploring the importance of cueing goal-oriented actions and training of social and adaptive skills that are increasingly being done in virtual environments (Simões et al., 2014, 2018).

Our present study emphasizes that these attentional allocation impairments are associated with EF deficits, which stresses a set of important questions we already raised, related not only to the school intervention, but also to full social inclusion of ASD young adults in a society that is highly competitive and requires so many social and EF abilities. Additionally, notwithstanding the superior or average IQ, subjects with ASD experience substantial difficulties in everyday life (Mouga et al., 2015, 2016), which can lead to an overvaluation of IQ in terms of predicting adaptive behavior skills and a misleading as good outcome without adequate assessment and consideration of EF and social skills.

Despite the fact that the use of virtual reality tasks in the clinical research has several gains compared to the real world settings, specifically in terms of affordability, safety, applicability, and efficiency of data collection (Parsons et al., 2017), we should have in mind that observed performance during simulated tasks may differ from what the individual does spontaneously in the real environment, since it is impossible to fully replicate the uncertainties of everyday life. And that could be pointed as one limitation of our study; however, we tried to simulate the aids that we could have in a supermarket, for instance, arrows and people helping us find what we need.

Another possible limitation of our study is that in the study of EF, it is difficult to isolate which specific type of EF is impaired and/or contributes to the performance deficits observed, as the tasks we used is complex and relies on multiple cognitive skills. Future studies should use a manipulation of the stimuli, in which specific hypotheses about the role of particular EF (for example, working memory, or inhibition), can be assessed.

The wide age range of our study constitutes another possible limitation. Future studies should focus on smaller age ranges.

In sum, our results emphasize important challenges in the overall attentional allocation in social/non-social cognitive processing in ASD, in the absence of overall quantitative intellectual disability. Intriguingly, social cognition impairment was further suggested by eye-tracking data on the social salient cues. The most consistent impairments in non-social cognition that we found were measured by the number of head rotations and enhanced by the increase of number of items, in the EcoSupermarketX. In fact, these were corroborated by the association of these parameters repeatedly with the Corsi Blocks and Tower in the ASD group. These tests evaluate EFs of planning, working memory, rule learning, the ability to inhibit responding, self-monitoring and regulation, and problem solving, as well as lower visuospatial short-term memory, which means that greater difficulties in our task were associated with difficulties in those areas. This provide us with the evidence that attention allocation and EF alterations may not only be a promising endophenotype for ASD, but also tasks that allow for evaluating these cognitive aspects which may have a determinant role in the differential diagnosis of subjects with ASD who do not have intellectual disability.

To extricate the association between attentional allocation, EF, and social vs. non-social cognition and to increase our understanding of the cognitive mechanisms of impairment in ASD, future studies need to continue to consider both non-social and social cognitive domains and focus on these domains in the study of the neural correlates of executive and attentional dysfunction in ASD.

## Data Availability Statement

The raw data supporting the conclusions of this article will be made available by the authors upon request, without undue reservation.

## Ethics Statement

All the procedures in this study were reviewed and approved by the ethics committees from Faculty of Medicine from University of Coimbra, Portugal (CE-11/2013) and the Centro Hospitalar e Universitário de Coimbra, Portugal (CHUC-102-13) and was conducted in accordance with the 1964 Helsinki declaration and its later amendments or comparable ethical standards. Written informed consent was obtained from the parents/guardians of all participants or, when appropriate, the participants themselves. Children and adolescents also gave oral informed consent.

## Author Contributions

SM, GO, and MC-B conceived and designed the study. ID programmed the task. SM performed the study and wrote the original manuscript. SM, CC, DS, and FD contributed with data collection. SM and ID analyzed the data. GO and MC-B performed supervision of all steps. ID, GO, and MC-B: reviewed and edited the manuscript. All authors read and approved the final manuscript.

## Conflict of Interest

The authors declare that the research was conducted in the absence of any commercial or financial relationships that could be construed as a potential conflict of interest.

## Publisher's Note

All claims expressed in this article are solely those of the authors and do not necessarily represent those of their affiliated organizations, or those of the publisher, the editors and the reviewers. Any product that may be evaluated in this article, or claim that may be made by its manufacturer, is not guaranteed or endorsed by the publisher.

## Abbreviations

ABC: autism behavior checklist
ADI-R: autism diagnostic interview—revised
ADI-R L/C: autism diagnostic interview—revised language/communication
ADI-R RB/I: ADI-R repetitive behaviors/interests
ADI-R RSI: ADI-R reciprocal social interactions
ADOS: autism diagnostic observation schedule
ADOS COM: ADOS communication
ADOS SI: ADOS social interaction
AOI: area of interest
ASD: autism spectrum disorder
BANC: Coimbra neuropsychological assessment battery
CA: chronological age
CHUC: Centro Hospitalar e Universitário De Coimbra
DSM: Diagnostic and Statistical Manual of Mental Disorders
EFs: executive functions
F: female
FSIQ: full-scale intelligence quotient
IQ: intelligence quotient
IQR: interquartile range
M: male
Max: maximum
Mdn: median
Min: minimum
PIQ: performance IQ
SCQ: social communication questionnaire
SCWT: Stroop color-word test
SPSS: statistical package for social sciences
SRS: social responsiveness scale
VIQ: verbal IQ.
